# Acute Effects of Different Electroacupuncture Point Combinations to Modulate the Gut-Brain Axis in the Minipig Model

**DOI:** 10.1155/2022/4384693

**Published:** 2022-10-20

**Authors:** Xuwen Zhang, Sylvie Guérin, Youenn Launay, Yann Serrand, Nicolas Coquery, David Val-Laillet

**Affiliations:** ^1^Guangzhou University of Chinese Medicine, Guangzhou, China; ^2^Guangzhou Rui Xingtang Chinese Medicine Clinic, Guangzhou, China; ^3^INRAE, INSERM, Univ Rennes, Nutrition Metabolisms and Cancer, NuMeCan, St Gilles, Rennes, France

## Abstract

This study aimed to compare the gut-brain axis responses to acute electroacupuncture (EA) at different acupoint combinations in the minipig model. Four adult Yucatan minipigs were subjected twice to four acute EA treatments (25-minute acute sessions) including sham (false acupoints) and control (no EA), during anesthesia and according to a Latin-square design paradigm. Acupoint combinations (4 loci each) are head-abdomen (#70 Dafengmen, #35 Sanwan), back (bilateral #27 Pishu, #28 Weishu), leg (bilateral #79 Hangou, #63 Housanli), and sham (2 bilateral points that are not acupoints). Electrocardiograms were performed to explore heart rate variability (HRV). Infrared thermography was used to measure skin temperature at the stimulation points. Saliva (cortisol) and blood samples (leptin, total/active ghrelin, insulin, and glucose) were collected for further analyses before and after acute EA. All animals were also subjected to BOLD fMRI to investigate the brain responses to EA. Acute EA significantly modulated several physiological and metabolic parameters compared to basal, sham, and/or control conditions, with contrasting effects in terms of BOLD responses in brain regions involved in the hedonic and cognitive control of food intake. The head-abdomen combination appeared to be the most promising combination in terms of brain modulation of the corticostriatal circuit, with upregulation of the dorsolateral prefrontal cortex, dorsal striatum, and anterior cingulate cortex. It also induced significantly lower plasma ghrelin levels compared to sham, suggesting anorectic effects, as well as no temperature drop at the stimulation site. This study opens the way to a further preclinical trial aimed at investigating chronic EA in obese minipigs.

## 1. Introduction

Traditional acupuncture and one of its modern derivatives, electroacupuncture (EA), are increasingly used for treating various diseases and especially gastrointestinal disorders [1, 2]. Acupuncture and EA are usually appreciated for their limited adverse effects and good clinical outcomes. Some evidence already suggested that EA, which consists in applying an electrical current stimulation on specific acupoints, provides better outcomes than acupuncture [3]. The gut-brain axis plays an important role in gastrointestinal functions and eating behavior control. The brain affects the digestive tract through the autonomic nervous system, by modulating regional gut motility and permeability, intestinal transit and secretion, and the secretion of hormones involved in metabolic homeostasis and food intake control [4]. Gut hormones or neuropeptides, such as ghrelin and CCK, can modulate gastrointestinal functions and transmit information to the brain through the blood system and the autonomic nervous system, especially the vagus nerve [5, 6]. Brain activity reflects and is influenced by eating patterns, food consumption, or cravings. The exploration of brain activity and responses, for example, *via* functional magnetic resonance imaging (fMRI), can provide crucial information about the sensory, cognitive, and hedonic integration of exteroceptive and interoceptive stimuli in healthy or pathological conditions [7]. It is a hot research topic for which rodent models have significant limitations and constraints, which justifies the use of large animal models when some scientific or methodological questions cannot be explored directly in humans. The present study is the first to investigate brain responses to acute EA in an animal model that is particularly interesting for nutrition and neurosciences research, the miniature pig also called minipig.

Many research studies from China or Western countries supported the beneficial effects of acupuncture or EA treatments on gastrointestinal regulation, through opioids, and other neural pathways [8, 9]. Even though the number of studies on acupuncture and EA increased significantly in the past decades, controlled mechanistic studies are scarce, and relevant preclinical studies lack support for the use of specific acupoint combinations that could be further tested in clinical trials to relieve gastrointestinal symptoms and modulate eating behavior. Most of the acupuncture and EA research studies were performed in rodent models, which present the advantage to be relatively cheap and easy to handle, with a low interindividual variability when it is required. Despite the proximity of this model to humans on many levels and its ease of use, the rodents' anatomy also has obvious limitations when it comes to identifying relevant acupoints for clinical practice. Moreover, their digestive system is very different from that of humans, and their small lissencephalic brain restrains significantly the field of possible research in neuroscience and neuroimaging. In our study, we chose to use the minipig model because this species presents many similarities with the human in terms of organ size and proportions, notably gastrointestinal tract anatomy, morphology, and physiology, besides having a quite large gyrencephalic brain better suited for brain functional imaging [10, 11]. Our INRAE research group gained renowned expertise on the minipig model for nutrition and neuroscience research and have implemented various *in vivo* brain imaging strategies to explore in pigs the brain activity and metabolism in response to olfactogustatory and other kinds of relevant stimulations [12–14] but also in the context of diet-induced obesity [11, 15]. In a recent review paper, we presented the obesity animal models for acupuncture and related therapy research studies [1]. The minipig's size and comparable anatomical structures present significant assets to explore the outcomes of acupoint or meridian stimulation as in humans [16, 17].

Single acupuncture points were often used in rodent experiments, but in clinical practice, a combination of different acupoints is usually recommended, and in Chinese medicine theory, point combinations provide better results than single-point stimulation thanks to synergetic effects. In our study, we chose three different acupoint combinations and a sham-point combination, as initially described in a previously published hypothesis paper [1]: a head-abdomen point combination associating Dafengmen (#70) and Sanwan (#35), a back point combination associating Liumai (#27) and Pishu (#28), and a leg point combination associating bilateral Housanli (#63) and Hangou (#79). The precise location of these pig acupoints and their correspondence with human acupoints will be described later. The difference between placebo and acupuncture, or between acupuncture points and nonacupuncture points, is not always clearly described in the literature [18, 19], and many studies did not include relevant control or sham treatment in their protocol. For our study, we chose two pairs of sham points that have not been identified as acupuncture points in the pig in order to discover whether differences could be observed between the stimulation of recognized acupoints and the stimulation of anatomical loci that are not relevant in the context of pig acupuncture.

In Chinese medicine theory, all the acupuncture points chosen for our study are classic acupoints that are commonly used in human clinical practice. They are thought to influence nutrition and digestive processes, notably by accelerating gut transit and water circulation in the body. Always according to the Chinese medicine theory, acupuncture points work in priority on surrounding organs. Modern research showed that EA at Zusanli (ST36) in conscious rats accelerated colonic motility and transit [20]. In contrast, another study showed that EA at Zusanli (ST36) inhibited colonic transit in restraint stress rats [21]. In Sprague-Dawley rats, Yang et al. [22] showed that both manual acupuncture (MA) and laser-acupuncture at Zusanli (ST36) increased gastric motility, while Zhongwan (CV12) suppressed gastric motility and Weishu (BL21) had no significant effect. Such discrepancies justify the need for more research and good controlled preclinical studies [23]. Our aim in this study was to compare, in a large animal model close to the human, different EA combinations by assessing their immediate effects on different parameters that are important for food intake control. In the hypothesis paper [1], we published preliminary to the preclinical trial presented here we identified several key markers relevant to investigate the gut-brain axis and food-brain cognition. Our hypothesis is that the EA combinations chosen for this preclinical trial will modulate differently from sham treatment the plasma level of gut hormones, glucose metabolism, autonomic nerve balance, and most importantly the brain responses concomitant to acute EA.

## 2. Materials and Methods

### 2.1. Animals

Experiments were conducted in accordance with the current ethical standards of the European Community (Directive 86/609/EEC), Agreement No. A35-622 (DVL), and Authorization No. 01894. The Regional Ethics Committee in Animal Experimentation of Brittany (n°007) has validated the entire procedure described in this paper (project n°APAFIS #21708-2019080810171624-V2) and the French Ministry of Higher Education, Research and Innovation authorized the whole project.

Four 18-month-old Yucatan minipigs (2 males and 2 females), weighing 52.18 ± 4.18 kg on average and coming from the INRAE minipig breeding facilities (UE3P, 35590 St Gilles, France), were used in this study. All pigs were fed a standard diet [24]. Animals were housed in individual pens (150 cm × 60 cm × 80 cm) and had free access to water and toys. The room was maintained at ∼24°C with a 13 : 11-hour light-dark cycle. Professional animal caretakers were in charge of surveying and handling the animals.

## 3. General Experimental Design

**Figure 1:**
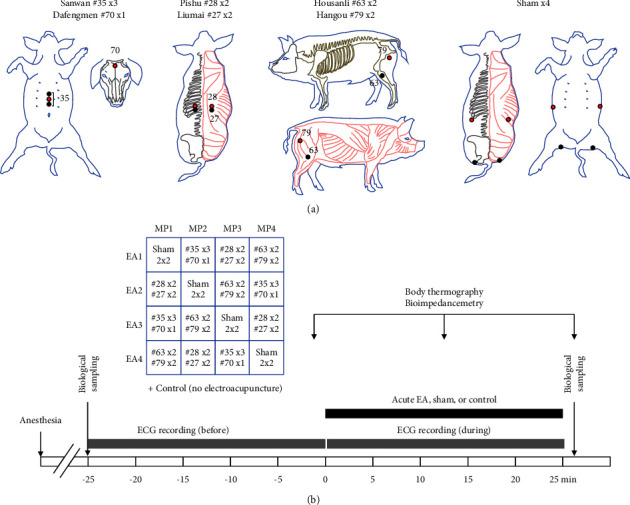
Schematic representations of the experimental design with (a) localization of the acupoints and sham points for electroacupuncture (EA), and (b) time course of each treatment session of acute electroacupuncture, sham stimulation, or control. Each animal (*N* = 4) was subjected to five different sessions (EA1, EA2, EA3, sham, and control). All acupoints were localized according to “Traditional Chinese Veterinary Acupuncture and Moxibustion” [25]. We selected three acupoint combinations as described in a previous hypothesis paper [1]. The head-abdomen point combination associated Dafengmen (#70) with Sanwan (#35). The back point combination included the bilateral Pishu (#28) as well as the bilateral third pair of pig acupoint Liumai (#27). The leg point combination associated the pig bilateral Housanli (#63) with the bilateral Hangou (#79). The sham combination included four loci that do not correspond to pig acupoints (two bilateral sham points on the back and buttock). Red points indicate cathodic stimulation and black points anodic stimulation. Control sessions were performed without any stimulation. The biological sampling included blood and saliva sampling. An electrocardiogram (ECG) was recorded for 25 min before and 25 min during EA or control treatment. Body thermography and bioimpedancemetry measurements were performed on three occasions just before, in the middle, and just after the EA or control treatment.

All minipigs were subjected twice (2 repetitions) to 4 different electroacupuncture (EA) sessions according to a Latin square (to randomize the treatment order amongst animals, Figure 1(a)) and one additional control session without EA stimulation, meaning that each animal underwent 10 experimental sessions, with at least 2 days between each session. All sessions were structured similarly (Figure 1(b)) and the acute treatment applied (EA, sham, or control) lasted 25 min. For each session, the animals were anesthetized and installed in a Pavlov stand equipped with a hammock, which standardized the body posture of the animals for all sessions while making each stimulation point accessible at all times. Blood and saliva samples were performed before and after each 25-minute EA, sham, or control session. Body infrared thermography (FLIR®Exx series, FLIR Systems, Inc, 2013) and bioimpedance measurements (RAPID Z-METRIX® and dedicated Z-Metrix Software, BioparHom, France) were performed before, in the middle, and after each session. Electrocardiogram (ECG) recordings were performed during a 25-minute period before and during the 25-minute EA, sham, or control sessions. A few days after the last experimental sessions, all animals were subjected to a brain imaging session using functional magnetic resonance imaging (fMRI) during which the 3 different EA treatments and sham procedures were performed in a randomized order in all animals. This fMRI session was performed at a temporal distance from the 10 previous interventions to limit their potential effect (including the effect of repeated anesthesia) on the brain responses to acute EA recorded on the day of imaging. At the end of the experiment, all the minipigs were euthanized with a lethal injection of T61 (1 mg/10 kg).

### 3.1. Acupuncture Points Selection and Location

All acupoints were localized according to “Traditional Chinese Veterinary Acupuncture and Moxibustion” [25]. We selected three acupoint combinations (Figure 1) as described in a previous hypothesis paper [1].

The head-abdomen point combination associated Dafengmen (#70), which is anatomically similar to human Baihui (GV20) on the head, with Sanwan (#35), similar to human Shangwan (RN13), Zhongwan (RN12), and Xiawan (RN10). Dafengmen (#70) is a single point located on the dorsal mid-sagittal plane between the two ear rostral bases. Sanwan (#35) is a three-locus acupoint located on the ventral midline of the abdominal region. The middle point is at the midpoint between the caudal end of the xiphoid process of the sternum and umbilicus. The cranial point is at the midpoint between the middle acupoint and the xiphoid process. The caudal point is at the midpoint between the middle acupoint and the umbilicus.

The back point combination included the bilateral Pishu (#28), corresponding to the second pair of point #27 similar to human Pishu (BL20), as well as the bilateral third pair of pig acupoint Liumai (#27), similar to human Weishu (BL21). Liumai (#27) is a three-locus acupoint located 6 cm lateral on each side of the dorsal midline, in the last three intercostal spaces, in the muscle groove of musculi longissimus dorsi and iliocostalis dorsi. The leg point combination is associated with the pig bilateral Housanli (#63), similar to human Zusanli (ST36), and the bilateral Hangou (#79), similar to human Huantiao (GB30).

The leg point combination associated the pig bilateral Housanli (#63), anatomically similar to human Zusanli (ST36), with the bilateral Hangou (#79), similar to human Huantiao (GB30). Housanli (#63) is located in the depression 6 cm caudoventral to the lateral edge of the patella, in the depression ventral to the head of the fibula, in the muscle groove of the long digital extensor, and in the lateral digital extensor. Hangou (#79) is located in the muscle groove between musculi biceps femoris and semitendinosus, ventral to the ischiatic tuberosity.

Sham points were nonacupoints. The first pair of sham points we chose were located bilaterally at the tip end of the fifteenth rib. The second pair of sham points were located bilaterally at the midpoint between the root end of the tail (1st caudal vertebra) and the musculus semitendinosus lower joint at the level of the depression visible above the tip end of the ossa tarsi. These anatomical loci were never associated with pig acupuncture in our knowledge and there is no equivalent either in human acupuncture.

### 3.2. Surgery for Implantation of a Venous Catheter

All animals were subjected to surgery a week before the beginning of the experiment in order to implant a venous catheter used for further blood sampling. Initial sedation was induced with an intramuscular injection of Zoletil®50—Virbac (1 ml/pig) after overnight fasting. Isoflurane inhalation (Aerane 100 ml, Baxter SAS, France) was used to suppress the pharyngotracheal reflex (3–5% during less than 5 minutes) before tracheal intubation. After intubation, a surgical level of anesthesia was maintained with inhaled isoflurane (2.5%, v/v) to achieve a 2.2 minimum alveolar concentration. Oxygen fraction (FiO2) and tidal volume were adjusted so that oxygen saturation (spCO2) measured by pulse oxymetry (Ohmedaoxymeter; GE Healthcare Clinical Systems, Limonest, France) was 98% or more and spCO2 measured by infrared capnometer (Armstrong capnometer; Gambo Engström, Bromma, Sweden) was <5%. Analgesia during surgery was achieved by a subcutaneous administration of fentanyl (30–100 mg/kg/h, i.v.; Renaudin, Paris, France). A silicon catheter was inserted into the left jugular vein, tunneled under the skin, and exteriorized on the top of the neck between the shoulders. Animals were allowed to recover for one week after the surgical procedure before any observation or treatment.

### 3.3. EA Treatment

#### 3.3.1. Anesthesia for EA

A similar anesthesia procedure as that described for surgery was used for the EA treatment, except that no analgesia was performed since EA is not a painful procedure. Every EA session lasted 25 minutes. A KWD-808I electric stimulator (Changzhou Yingdi Electronic Medical Devices Co. 414 Ltd), set at 2 Hz and 3 mA with continuous wave current, was used for every point combination to achieve local slight muscle contraction. Needles (Suzhou Acupuncture and Moxibustion Appliance Co, China) were 0.35*∗*40 mm for head point #70 (insertion depth of about 4 mm), 0.35*∗*40 mm for back points #27 and #28 (insertion depth of about 20 mm), 0.35*∗*75 mm for abdomen point # 35 and leg points #79 and #63 (insertion depth of about 40 mm), and 0.35*∗*40 mm for sham points (insertion depth of about 20 mm). Cotton wool was used to conceal the animal's ears, and the tape was used to maintain the eyes closed to decrease exteroceptive sensory stimulation during the treatment.

### 3.4. Heart Rate Variability

Heart rate variability (HRV) was calculated as LF (low frequency)/HF (high frequency). HRV was analyzed from electrocardiographic recordings obtained using II derivation [26]. Briefly, the electrocardiogram (ECG) was recorded using an ECG machine (GE, USA) in a driven right leg configuration and digitized at 400 Hz (NI-USB-6008, National instruments, USA) after low-pass filtration (analog Butterworth filter set at 200 Hz) to cancel aliasing. The digitized signal was streamed continuously using TDMS format and analyzed afterward with Labview biomedical toolkit (National instruments, USA). After QRS recognition, the RR intervals were detrended and analyzed in the frequency domain with an autoregressive algorithm to extract low frequencies (LF—0.04 to 0.15 Hz) and high frequencies (HF—0.15 to 0.40 Hz) components as well as the ratio of both. LF and HF were normalized with the total power of the autoregressive spectrum between 0 and 0.4 Hz (1996). Measurements were performed on anesthetized animals for 25 minutes before the EA session as a baseline and then again for 25 minutes during the EA session.

### 3.5. Acupuncture and Sham Points Temperature

FLIR®Exx series (FLIR Systems, Inc, 2013) was used to take infrared thermal and normal-light standard pictures of acupoints. This device was operated by the same person (XZ) during the whole experiment, at a 1-meter distance from the acupoint, with the same angle of view for each session to reduce the risk of bias. Before and after every EA session, three repeated measures were performed at each acupoint to get stable averaged data.

#### 3.5.1. Body Bioimpedance Measurements

Body bioimpedance measurements (RAPID Z-METRIX®) were performed before, in the middle, and after each EA and control session. Data were extracted from the automated analysis performed by the dedicated Z-Metric Software (BioparHom, France). These data included reactance and resistance values at 1, 5, 50, 150, 200, 250, and 325 kHz, as well as several estimated physiological parameters including metabolic activity index, fat mass, nonfat mass, muscle mass, body protein composition, and mineral bone composition, for example. However, these estimated parameters were calculated upon human equations that do not fit with the minipig model, which limits considerably their interpretation.

### 3.6. Biochemical Analyses

Saliva and blood samples were obtained from anesthetized animals before and after every EA session. Pig saliva collection was done before intubation anesthesia by a dedicated saliva sampling kit for further cortisol analyses. Blood samples were taken from the jugular catheter. Blood and saliva samples were immediately centrifugated at 4°C for 10 minutes. Plasma for glucose, insulin, leptin, and total ghrelin assays was stored at −20°C. Plasma for active ghrelin assays was frozen in liquid nitrogen before being stored at −80°C. Saliva supernatant for cortisol assays was stored at −20°C. Salivary cortisol level was assessed by the immunoluminescence method, glucose levels by the enzymatic method, insulin levels by the immunoenzymatic method, and ghrelin levels by the radio-immunologic labeling method.

*Salivary Samples and Analyses.* Pigs had to chew cotton buds (SalivetteR, Sarstedt, Nümbrecht, Germany) for 1 min. Buds were rapidly centrifuged (2,500 G, 10 min, 4°C), and supernatants were stored at −20°C. Cortisol was quantified with a luminescence immunoassay kit (IBL, Hamburg, Germany) and read with a luminometer (Mitras LB940, Bertold Technologies).

*Blood Samples and Analyses.* Samples were collected from the jugular vein into EDTA, dry tubes (Vacutest Kima, Arzergrande, Italy), or aprotinin (BD LifeScience, Le Pont-de-Claix Cedex, France) vacutainers. Samples were kept in ice until centrifugation (2,500 G, 10 min, 4°C), and plasma was stored at −20°C or −80°C (according to kit specifications) until further analyses. Glucose was assessed by an automated spectrophotometric method (Konelab 20i, Thermo FisherScientific, Illkirch, France) using a specific kit. Insulin concentrations were obtained using immunoenzymologic kit (AIA-1800 Tosho Europe, Tessenderlo, Belgique) using a specific kit: ST AIA-PACK IRI. The Homeostatic Model Assessment for Insulin Resistance (HOMA-IR) index was calculated on the basis of glucose and insulin data [27]. Leptin concentrations were obtained using a radioimmunoassay kit (Millipore multispecies leptin RIA) [28]. Total ghrelin concentrations were obtained using a radioimmunoassay kit (MI-GHRT-89HK, IDS France, immunodiagnostic systems, Pouilly-en-Auxois, France). Active ghrelin concentrations were obtained using a radioimmunoassay kit (MI-GHRA-88HK, IDS France, immunodiagnostic systems, Pouilly-en-Auxois, France).

### 3.7. Functional Imaging

#### 3.7.1. Anesthesia

A similar anesthesia procedure as that described for EA sessions was used for brain imaging, except that anesthesia was maintained with a magnetic-compliant respiratory system (Fabius, Draeger, Germany). Heart rate was always comprised of a range between 80 and 130 beats per minute. Animals were covered with a blanket during imaging but the body temperature was not recorded. Cotton wool and an additional headset were used to conceal the animal's ears, and the tape was used to maintain the eyes closed in order to limit auditory and visual stimulation during the procedure.

### 3.8. Stimulation Paradigm

All four animals were subjected to the four EA treatments previously described (head-abdomen, back, leg, and sham combinations), of which the order was randomized. For each EA treatment, the sequence of stimulation consisted of the alternation between EA stimulation (ON: 30 sec) and rest (OFF: 30 sec), repeated 20 times. The purpose was to investigate the immediate brain responses to each of the four acute EA treatments.

### 3.9. MRI Image Acquisition

Image acquisition was performed as previously described [12, 13] on a 1.5-T magnet (Siemens Avanto) at the Rennes Platform for Multimodal Imaging and Spectroscopy (PRISM AgroScans, Rennes, France). Acquisitions were performed using a combination of coils (body and spine surface matrix coils, commercial products from Siemens, 6 channels were used for each) for optimized signal-to-noise ratio acquisition. Gradient shimming was performed automatically.

*T1 Weighted Anatomical Image Acquisition*. An MP-RAGE sequence was adapted to the adult minipig anatomy (160 slices, 1.2 × 1.2 × 1.2 mm^3^, NA = 2, TR = 2400 ms, TE = 3.62 ms, TI = 854 ms, FA = 8°, acquisition duration 15 min).

*BOLD (Blood-Oxygen-Level Dependent) Signal Acquisition*. An echo planar imaging sequence was adapted to pig head geometry (32 slices, TR/TE: 2500/40 ms, FA: 90°, voxel size: 2.5 × 2.5 × 2.5 mm^3^). The field of view was 180 × 180 mm, the matrix size was 64^2^, and the total EPI imaging time was 20 min 30 (492 volumes *x* 2.5 seconds/volume, 4 initial volumes as dummy scans). The ?rst four acquired volumes were excluded from the data analysis, meaning that no stimulation was performed during this period.

### 3.10. Data Analysis and Statistical Image Analysis

Data analysis was performed with SPM12 (version 6906, Wellcome Department of Cognitive Neurology, London, UK). After slice timing correction, realignment, and spatial normalization on a pig brain Atlas (41), images were smoothed with a Gaussian kernel of 4 mm. Due to limitations related to the size of the pig brain and the effect of anesthesia on brain activity, we used a nonstandard statistical analysis with regard to human statistical standards usually considering statistical significance at a cluster level with *p* value < 0.05 under FDR correction. Further details regarding the validity and limitations of the statistical approach used in this model and paradigm are developed in a previous paper from our team [12].

*Voxel-Based Statistic*. First-level (within-individual contrast) and second-level (within-group contrast) statistics were assessed with a threshold set at *P* < 0.05 to produce the brain maps of activation.

*SVC-Based Statistics (Small Volume Correction)*. Twenty-eight anatomical regions of interest (ROIs) corresponding to 14 bilateral structures were used: nucleus accumbens (Acc), caudate nucleus (Cd), putamen (Pu), globus pallidus (GP), parahippocampal cortex (PHC), hippocampus (Hi), insular cortex (Ins), orbitofrontal cortex (OFC), anterior and dorsolateral PFC (aPFC and dlPFC), ventral and dorsal anterior cingulate cortex (vaCC and daCC), and ventral and dorsal posterior cingulate cortex (vpCC and dpCC). The choice of these ROIs was made in our previous work in the context of food intake control and obesity in the minipig model [15, 24, 29, 30]. They were studied with a *p* value corrected with a Bonferroni correction at a threshold of 0.01 (peak level). The related uncorrected *p* value threshold after Bonferroni correction was consequently 0.00036.

### 3.11. Other Data Analysis and Statistical Image Analysis

All statistical analyses were performed using SPSS version 20 (SPSS, Chicago, IL, USA) with the alpha level set at *p* < 0.05. The analyzed data with normal distribution and variance homogeneity included body temperature and bioimpedance, salivary cortisol, glucose and insulin plasma levels, and heart rate variability (HRV). ANOVAs followed by LSD tests were applied to compare the treatments at each time point (before and after), and paired *t*-tests were applied to compare within treatments the data before (baseline) and after treatment.

## 4. Results

### 4.1. Heart Rate Variability (HRV)

Acute EA stimulation at back acupoints significantly decreased the LF/HF ratio in comparison to baseline (*t* = 2.38; *P*=0.049), and there was no difference for the other treatments (Table 1). After acute EA stimulation, the LF/HF ratio was higher with head-abdomen acupoints compared to leg acupoints (*F* = 1.447; *P* < 0.049), with no other group differences.

### 4.2. Body, Acupuncture, and Sham Points Temperature

Body temperature decreased after all interventions (*P* ≤ 0.001 for all), and there were no differences between groups (Table 1). Before acute EA, skin temperature at head-abdomen acupoints was significantly lower than at back and leg acupoints (*F* = 7.91; *P* ≤ 0.001 for both). After acute EA stimulations, head-abdomen acupoints showed a lower skin temperature compared to the leg, back, and sham stimulation points (*F* = 5.773; *P*=0.023, *P* < 0.001 and *P*=0.049, respectively), and there were no other differences between groups (*P* > 0.10). Skin temperature recorded after acute EA was lower than before for the back, leg, and sham points (*P* < 0.001 for back and leg, *P*=0.012 for sham). The minus value ‘after-before' was also lower in the sham compared to the leg (*F* = 1.372; *P*=0.043).

### 4.3. Body Bioimpedance Measurements

Reactance at 5 kHz was significantly decreased after versus before acute EA stimulation at head-abdomen only, whereas reactance at 325 kHz significantly increased in the sham group (*P* < 0.05 for both). There were no other differences in reactance and resistance data at any frequency. Metabolic activity index, nonfat mass, muscle mass, and body protein composition did not differ between treatments (*P* > 0.10). Leg acute EA treatment showed a significant increase (middle and after versus before) in fat mass and a decrease (middle versus before) in mineral bone composition (*P* < 0.05 for all). However, it is necessary to remind here the limitation of these measurements since they are extrapolated to minipigs on the basis of human-based equations.

### 4.4. Saliva and Plasma Analyses

All data are synthetized in Table 1.

*Cortisol (Saliva)*. There was no difference between groups but a significant increase in plasma cortisol after treatment compared to before all treatments (*P*=0.048 for Sham, *P* < 0.01 for other comparisons, with a *t*-value varying from −2.39 to −5.19).

*Leptin (Plasma)*. All groups except for back (*P* > 0.10) acupoints showed a significant decrease in plasma leptin after treatment compared to before (*P* < 0.05 for the other comparisons), and there were no differences between groups before or after treatment.

*Ghrelin (Plasma)*. After treatment, total ghrelin was lower for the head-abdomen compared to sham EA (*F* = 1.622, *P*=0.021), and there were no other differences. For plasma active ghrelin, there were no differences between groups, either before or after acute EA. However, plasma active ghrelin significantly increased after all interventions (*P* < 0.05 for all).

*Insulin (Plasma).* Except for Leg EA (*P* > 0.10), plasma insulin levels decreased after all treatments (*P*=0.012 for control, *P* ≤ 0.001 for the other treatments, with t-values from 3.38 to 8.52). There were no significant differences between treatments for raw insulin data. However, the ‘after minus before' values for insulin levels were significantly lower for the back and leg compared to the control (F = 1.98; *P*=0.025 for the back, *P*=0.032 for the leg).

*Glucose (Plasma).* There were no differences in plasma glucose between treatments nor between time points within treatments, except after sham treatment compared to before (*t* = 2.63, *P*=0.034).

*HOMA-IR (plasma)*. Back and head-abdomen EA presented lower HOMA-IR compared to control before the intervention, while leg EA presented higher HOMA-IR compared to sham after intervention (*P* < 0.05 for all). All interventions except for leg EA induced a significant reduction of HOMA-IR over time (*P* < 0.05 for all). The ‘after minus before' HOMA-IR value was lower in control compared to back and leg EA.

### 4.5. Functional Magnetic Resonance Imaging (fMRI) Analyses

**Figure 2:**
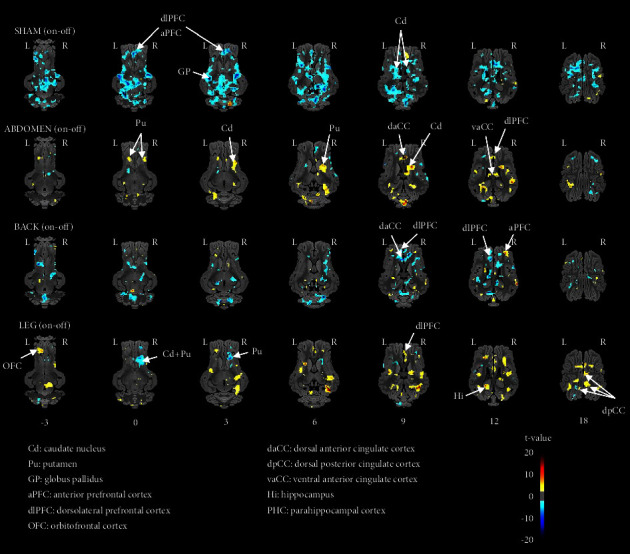
Horizontal maps of brain blood-oxygen-level-dependent (BOLD) functional magnetic resonance imaging (fMRI) responses of minipigs to four types of electroacupuncture (EA) stimulation compared to control (no stimulation): sham (two bilateral sham points on the back and buttock), head-abdomen (cephalic Dafengmen #70 associated with abdominal three-locus Sanwan #35), back (bilateral Pishu #28 and Liumai #27), and leg (bilateral Housanli #63 and Hangou #79). The significance threshold was set at *p*=0.05, *k* > 20. The coordinates in the dorsal-ventral position related to the posterior commissure (in mm) are indicated below each slice level. Positive *t*-values (hot colors) indicate higher activation in the four EA groups compared to the control, whereas negative *t*-values (cold colors) indicate lower activation in the four EA groups compared to the control.

As illustrated on the brain activation maps (Figure 2), the sham EA stimulation paradigm, as determined with the on-off contrast (off meaning no EA stimulation, i.e., control), elicited mainly lower brain responses in a large number of brain regions including striatopallidal ganglia (e.g., Cd and GP) associated with reward/cognition/emotion/motor responses, and prefrontal regions associated with cognitive functions, inhibitory control, and decision-making (aPFC and dlPFC). On the contrary, the head-abdomen EA stimulation paradigm promoted higher brain responses in the reward and motivational centers (Cd, Pu), in brain regions involved in associative learning and emotions (daCC and vaCC), and in the dlPFC, which is involved in decision-making. The back EA stimulation paradigm promoted contrasted brain responses, with higher activation in the aPFC and lower activation in dlPFC and daCC. The leg EA stimulation paradigm also elicited contrasted brain responses, with higher activation in prefrontal regions associated with cognitive functions, decision-making and/or hedonic valuation (dlPFC and OFC), and lower brain activation in brain regions including the reward and motivational centers (Cd and Pu).

*Corrected SVC-Based Statistic* (Table 2). The Sham EA stimulation paradigm promoted only lower activation (on-off contrast) in nearly all brain regions included in the SVC-based analysis. The back EA stimulation paradigm also promoted lower brain activation but only in the right daCC (*p*_uncor_ = 0.00002) and in the right dpCC (*p*_uncor_ = 0.00003). The abdomen EA stimulation paradigm elicited brain activation in the left daCC (*p*_uncor_ = 0.00002; *p*_FWE_ = 0.0084) and in the right vaCC (*p*_uncor_ = 0.0003; *p*_FWE_ = 0.0184). The leg EA stimulation paradigm promoted higher brain activation in the left dpCC (*p*_uncor_ = 0.00004; *p*_FWE_ = 0.0327) and in the left PHC (*p*_uncor_ = 0.0003).

**Figure 3:**
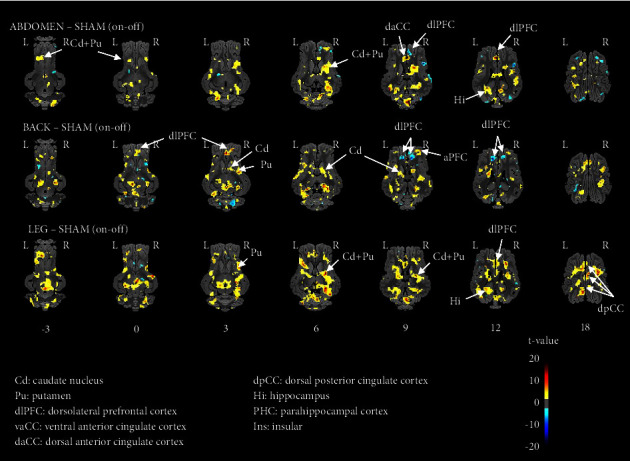
Horizontal maps of brain blood-oxygen-level-dependent (BOLD) functional magnetic resonance imaging (fMRI) responses of minipigs to the three types of electroacupuncture (EA) stimulation compared to sham (two bilateral sham points on the back and buttock; i.e., points that are not acupoints): head-abdomen (cephalic Dafengmen #70 associated with abdominal three-locus Sanwan #35), back (bilateral Pishu #28 and Liumai #27), and leg (bilateral Housanli #63 and Hangou #79). The significance threshold was set at *p*=0.05, *k* > 20. The coordinates in the dorsal-ventral position related to the posterior commissure (in mm) are indicated below each slice level. Positive *t*-values (hot colors) indicate higher activation in the three EA groups compared to the sham, whereas negative *t*-values (cold colors) indicate lower activation in the three EA groups compared to the sham.

Compared to sham EA, all three EA stimulation paradigms elicited, with some exceptions, higher brain responses as illustrated on the brain activation maps (Figure 3). Compared to the sham, the abdomen EA stimulation paradigm promoted higher brain responses in the reward and motivational centers (Cd, Pu), in memory and associative learning brain regions (Hi and daCC). A contrasted brain response with either higher or lower brain activation was detected in the dlPFC. Compared to the sham, the back EA and the leg EA stimulation paradigms yielded similar brain responses as observed with the abdomen EA stimulation paradigm but with some distinctions: the back EA stimulation paradigm promoted a higher brain response in the aPFC and no higher activation in Hi, whereas the leg EA stimulation paradigm promoted only higher brain responses in the dlPFC.

*Corrected SVC-Based Statistic* (Table 3). Overall, the three EA stimulation paradigms promoted higher brain activation compared to the sham. Compared to the sham, the abdomen EA stimulation paradigm elicited higher brain activation in the Pu (bilateral, left: *p*_uncor_ = 0.00036, right: *p*_uncor_ = 0.0001), right Hi (*p*_uncor_ = 0.0002), right daCC (*p*_uncor_ = 0.00004), and right PHC (*p*_uncor_ = 0.00001). Compared to the sham, the back EA stimulation paradigm promoted higher brain activation in the left Cd (*p*_uncor_ = 0.00004), right Ins (*p*_uncor_ = 0.0002), right daCC (*p*_uncor_ = 0.00001), left dpCC (*p*_uncor_ = 0.00007), and the right PHC (*p*_uncor_ = 0.00001) but also lower brain activation in the right dlPFC (*p*_uncor_ = 0.000035). Compared to the sham, the leg EA stimulation paradigm promoted higher brain activation in the right Cd (*p*_uncor_ = 0.0001), left Pu (*p*_uncor_ = 0.0002), Hi (bilateral, left: *p*_uncor_ = 0.0001; right: *p*_uncor_ = 0.00003), and dpCC (bilateral, left: *p*_uncor_ = 0.0002; right: *p*_uncor_ = 0.0001).

## 5. Discussion

This study is the first to implement electroacupuncture (EA) in the minipig model with the aim of modulating gut-brain signals and the BOLD fMRI hemodynamic responses in brain regions involved in food intake control, pleasure, and motivation. In a previously published hypothesis paper [1], we identified three acupoint combinations likely to have an effect on the gut-brain axis, including changes in the autonomic nervous system and especially the sympathovagal balance, as well as of hunger and satiety plasma markers. The present princeps study demonstrated that acute EA on specific acupoints significantly modulated several of these physiological and metabolic parameters compared to basal, sham, and/or control stimulation, with contrasting effects in terms of BOLD responses in brain regions involved in the hedonic and cognitive control of food intake. The head-abdomen combination (single acupoint Dafengmen #70 with three-locus Sanwan #35) appeared to be the most promising combination in terms of brain modulation of the corticostriatal circuit (compared to control and sham). It was also the only treatment that induced significantly lower plasma ghrelin levels compared to sham, suggesting anorectic effects, as well as no temperature drop at the stimulation site, suggesting thermogenesis effects. Different EA combinations had different outcomes on the heart rate variability, back EA being the only treatment that significantly decreased the LF/HF ratio. The back and leg treatments also induced a higher decrease in plasma insulin levels compared to the control, and the temperature decrease at the stimulation points was significantly higher for the leg compared to the sham. Even though the decrease in HOMA-IR was higher in control and sham interventions compared to EA at acupoints, these data are difficult to interpret especially because differences between groups were already observed before intervention.

As advocated in our recent hypothesis and review papers [1,2], the pig, especially the minipig, represents an excellent animal model to investigate the biological mechanisms related to acupuncture and related therapies such as EA. Most of the preclinical studies focusing on this thematic used rodent models, of which the size is very small and for which anatomical landmarks are very different from those used in the human, which complicates the transposition of acupoints localization. A large animal model such as the pig, presenting many anatomical and physiological analogies with the human, represents a real asset for translational research. Moreover, because of its economic importance in the context of livestock farming, the pig benefited from dedicated research on acupuncture and traditional veterinary medicine [25]. The existing textbook on “Traditional Chinese Veterinary Acupuncture and Moxibustion” provides a solid foundation to identify and localize acupoints in this species [25]. Finally, the large gyrencephalic brain of the pig, in comparison to the small lissencephalic brain of rodents, makes the use of brain imaging modalities and machines similar to those used in humans possible. Functional brain imaging has already been used to investigate the effects of different types of food sensory stimulations, diets, and obesity treatments on brain functions and metabolism in anesthetized pigs [11]. Anesthesia certainly modifies the body and brain responses to EA compared to what might be induced in awake individuals, but it is a stressless and mandatory procedure to make sure that animals do not move during imaging. According to traditional Chinese medicine, De-Qi experienced by patients is often described as as suan (aching or soreness), ma (numbness or tingling), zhang (fullness, distention, or pressure), and zhong (heaviness) and is felt by the acupuncturists (needle grasping) as tense, tight, and full [19]. Of course, anesthetized animals cannot consciously perceive these sensations and even less communicate about them, contrary to nonanesthetized human patients. This does not mean, however, that there is no feedback since the acupuncturist can still perceive the twitching of local muscles during EA in anesthetized pigs and feel De-Qi in his/her fingers when inserting and adjusting the needles.

A preclinical study in rabbits demonstrated that EA induced thermal changes in soft tissues, with initial and transient vasoconstriction at the negative pole and vasodilatation at the positive pole, followed by a slight temperature increase at both poles that persisted until the end of treatment [31]. In human patients suffering from acute lumbar muscle sprain, Fan and Wu [32] showed that EA at bilateral Jouxi, Jiaji, and Ashi points significantly increased lumbar skin temperature with greater recovery assessed through infrared thermograms compared with the medication group (diclofenac sodium). In our study, the overall body temperature decrease in all treatments was easily explained by the effects of anesthesia (body temperature was already below normal values before treatment), and the initial difference in temperature between acupoints is also logical because skin surface temperature differs according to body parts rather than meridians, as previously documented [33]. We did not find any skin temperature increase during and immediately after the 25-minute acute EA treatments in our animals. Acupoint skin temperature was significantly decreased in all EA treatments, except for the head-abdomen combination, possibly suggesting thermogenesis. Slight local muscle contractions observed during EA (for all treatments) should rather induce a mild increase in surface temperature, but the general decrease in body temperature probably antagonized this effect. Local vasoconstriction might also explain a local temperature decrease, which is known to be under the control of the sympathovagal balance.

The only EA treatment that significantly impacted the heart rate variability (HRV) was that performed at the back combination, with a significant decrease of the low-frequency/high-frequency (LF/HF) ratio compared to basal, but there were no differences compared to sham and control. Heart rate variability (HRV) can provide indirect insight into the autonomic nervous system activity and consequently into sympathetic and vagus tones [34], even though the reliability of this proxy is debated [35]. Some studies showed decreased HRV in patients with autonomic function problems or type-2 diabetes with autonomic neuropathy compared to normal subjects [36]. Many authors suggest that the ratio of LF to HF reflects the sympathovagal balance and that an increased LF/HF ratio indicates low vagal activation [37,38]. Our results might rather support a possible vagal activation, which would not concur with the hypothesis of a sympathetic activity-mediated local vasoconstriction [39]. Why this effect was observed for the back combination only cannot be explained without further investigations, but variability between loci in terms of needle insertion depth, local muscle contractility, or anatomical proximity with nerve bundles might contribute to differences between treatments.

Hormones such as ghrelin, leptin, and insulin play an important role in gut-brain communication, transmitting signals via vagal afferents or the blood system notably, from endocrine organs to brain regions regulating eating behaviors through appetite, hunger, and reward [40, 41]. We found after acute EA stimulation at head-abdomen acupoints that plasma total ghrelin levels were lower than those after EA at sham points but there were no group differences for active ghrelin (increasing after all treatments including control), which limits the interpretation of this result. All treatments except for EA at back acupoints decreased plasma leptin levels in comparison to basal measurements, which is coherent with previous studies in humans [42–45]. However, we cannot discard a potential interaction between treatment and anesthesia in our study, since previous studies demonstrated that isoflurane anesthesia can alter blood levels of endocrine hormones and increase stress [46], as illustrated by the cortisol increase in all our conditions. Another hypothesis is that a general anesthesia-induced decrease in leptin levels was counteracted by acute EA at back acupoints. Plasma insulin levels decreased in all EA treatments except for leg acupoints and the insulin drop (after-before) was higher in control than in the back and leg treatments, which is surprising considering that decreased insulin levels and insulin resistance were demonstrated in obese women [43] and rats [47] after EA on limbs. It is important to remind here that the minipigs used for this princeps study had a normal body weight, hormone variations were observed during anesthesia after short-term acute EA only, and the insulin response of normal-weight minipigs was shown to be different from that of obese subjects with basal hyperinsulinemia, higher insulin excursions, and insulin resistance [48,49]. Our results consequently do not presage what the effects of chronic EA would be in obese nonanesthetized minipigs, which will be the purpose of a further study.

Research studies about acupuncture or EA effects on brain activity are still very scarce, and selected acupoints in published papers are limited. Acupuncture at Zusanli (ST36) and Yinlingquan (SP9) (leg acupoints) in overweight Chinese men increased hypothalamus (HYP)-hippocampus functional connectivity (FC), HYP-putamen-insula FC, and HYP-anterior cingulate cortex FC, which were correlated with core body temperature, glucose, and hunger responses, respectively [50]. The authors suggested that increased dopamine modulation during acupuncture was most likely associated with the decreased poststimulation limbic system. Interestingly, Napadow et al. [51] showed that acupuncture at bilateral Zusanli (ST36) in healthy right-handed subjects induced BOLD fMRI signal increase in the anterior insula and decrease in limbic and paralimbic areas including the amygdala, anterior hippocampus, and ventromedial prefrontal cortex, notably. Finally, only EA activated the anterior middle cingulate cortex (CC) [51]. This is consistent with our own results demonstrating that the head-abdomen and leg EA stimulation induced activation in the prefrontal cortex (PFC), dorsal striatum, and CC. Only head-abdomen activated the dorsal anterior cingulate cortex whereas the leg activated the hippocampus. Brain responses with back EA were more ambivalent and difficult to interpret, but a major highlight of our study is that all three EAs at recognized acupoints in the pig produced activation of the frontostriatal and limbic circuits compared to sham EA, which rather induced widespread deactivation compared to control (ON-OFF).

One hypothesis about subjects presenting anomalies of the frontostriatal circuit, as described in obese humans and minipigs [7, 15, 52], is that a reward deficit associated with lower striatal responses is combined with decreased inhibitory control associated with lower dorsolateral PFC (dlPFC) responses. To counteract or compensate for these functional anomalies, an effective treatment might be aimed at increasing brain activity in these specific regions. For example, Ray et al. [53] investigated the effect of transcranial direct current stimulation (tDCS) on the dorsolateral prefrontal cortex to treat eating disorders and obesity. Unfortunately, they showed that participants who were told they were receiving real tDCS craved and ate less than participants told they were receiving tDCS, regardless of the tDCS condition administered. This demonstrates the powerful effects of expectation and autosuggestion (i.e., placebo effect), as well as the necessity to provide good control conditions in research. Other authors showed that neurofeedback training (i.e., a type of biofeedback that presents real-time feedback from brain activity in order to reinforce healthy brain function through operant conditioning) successfully helped patients to upregulate their dlPFC and decrease their palatability and choice ratings for chocolate cookies [54]. Our results in the minipig model demonstrated that EA at both head-abdomen and leg acupoints successfully increased the activity of the dfPFC compared to sham, with no possibility of the placebo effect in this model.

Our main goal for this princeps study was to select one promising acupoint combination for a preclinical trial in obese minipigs subjected to chronic EA. Brain activity modulations were quite comparable between the head-abdomen and leg, but the leg combination produced major deactivations in the dorsal striatum contrary to the head-abdomen that induced upregulation of this brain area involved in pleasure. Lower basal activity in the dorsal striatum has been identified as a component of a reward deficit that might favor increased food intake and weight gain as stated by the reward deficit theory [55], even though this theory is now debated [56]. Also, EA at head-abdomen upregulated the activity of the anterior cingulate cortex, contrary to the leg, which is an important choice criterion since the anterior CC is involved in conflict monitoring and in the cognitive inhibition network [57].

Altogether, our results provided a successful comparison between several acupoint combinations of interest for EA in a pertinent preclinical animal model. Even though the animal number was low, significant differences in the gut-brain axis responses were recorded between treatments, with EA at head-abdomen acupoints (Dafengmen-Sanwan) leading to upregulation of the corticostriatal circuit involved in hedonism and cognitive inhibitory control of eating, decreased ghrelin plasma levels, and different local thermal responses at the site of stimulation. This princeps study was a prerequisite for a further preclinical trial aiming at unraveling the effects of chronic EA in obese minipigs to decrease food intake and weight gain. This work will be valuable to explore the mechanisms of EA as a treatment for obesity or eating disorders, before its implementation in clinical trials with human patients.

## Figures and Tables

**Table 1:** Physiological responses of adult minipigs to four types of electroacupuncture (EA) stimulation compared to control (no stimulation): head-abdomen (cephalic Dafengmen #70 associated with abdominal three-locus Sanwan #35), back (bilateral Pishu #28 and Liumai #27), leg (bilateral Housanli #63 and Hangou #79), and sham (two bilateral sham points on the back and buttock). LF/HF: low frequency/high frequency. The duration of each treatment session was 25 min and all animals were subjected twice to all five treatments on separate days according to a Latin-square design.

	Head-abdomen acupoints		Back acupoints		Leg acupoints		Sham points		Control (no EA)	
	Before EA	After EA	Before EA	After EA	Before EA	After EA	Before EA	After EA	Before control	After control
Heart rate variability (ratio LF/HF)	0.282 ± 0.24	0.191 ± 0.19 a	0.170 ± 0.18	0.070 ± 0.07	0.206 ± 0.18	0.065 ± 0.05 b	0.244 ± 0.11	0.140 ± 0.11	0.108 ± 0.09	0.095 ± 0.12
minus value (after-before)	−0.091 ± 0.389		−0.101 ± 0.114^*∗*^		−0.141 ± 0.207		−0.105 ± 0.149		−0.014 ± 0.042	
Body temperature (°C)	34.98 ± 0.92	34.15 ± 0.86	35.40 ± 0.86	34.57 ± 0.83	35.25 ± 0.42	34.40 ± 0.67	35.38 ± 0.40	34.56 ± 0.32	35.38 ± 0.61	34.66 ± 0.61
minus value (after-before)	−0.83 ± 0.16^*∗*^		−0.83 ± 0.28^*∗*^		−0.85 ± 0.41^*∗*^		−0.82 ± 0.19^*∗*^		−0.74 ± 0.31^*∗*^	
Point stimulation (°C)	31.39 ± 1.58 b	30.99 ± 1.30 b	32.61 ± 0.78 a	32.05 ± 0.83a	32.37 ± 1.01a	31.58 ± 1.12 a	31.79 ± 0.92	31.50 ± 0.75 a	No EA point for control	
minus value (after-before)	−0.40 ± 1.71		−0.56 ± 0.56^*∗*^		−0.79 ± 0.82^*∗*^		−0.29 ± 0.61^*∗*^		—	
Saliva cortisol (ng/ml)	0.68 ± 0.54	1.88 ± 0.97	0.86 ± 0.56	2.02 ± 0.61	0.64 ± 0.50	1.40 ± 0.37	0.57 ± 0.45	1.44 ± 0.64	0.68 ± 0.41	2.08 ± 1.24
minus value (after-before)	+1.20 ± 0.88^*∗*^		+1.17 ± 0.86^*∗*^		+0.73 ± 0.62^*∗*^		+0.87 ± 0.47^*∗*^		+1.40 ± 1.26^*∗*^	
Plasma analyses										
Leptin (ng/ml)	4.77 ± 2.33	3.32 ± 1.06	4.59 ± 1.55	3.62 ± 1.21	5.18 ± 2.49	3.96 ± 2.19	5.42 ± 2.02	4.18 ± 0.95	5.55 ± 2.18	4.47 ± 2.14
minus value (after-before)	−1.44 ± 1.62^*∗*^		−0.93 ± 1.51		−1.22 ± 1.19^*∗*^		−1.24 ± 1.45^*∗*^		−1.07 ± 1.05^*∗*^	
Total ghrelin (pg/ml)	831 ± 409	580 ± 57 b	658 ± 63	640 ± 59	658 ± 96	594 ± 69	843 ± 529	737 ± 291 a	986 ± 736	673 ± 46
minus value (after-before)	−251 ± 437		−18 ± 81		−63 ± 123		−106 ± 646		−314 ± 709	
Active ghrelin (pg/ml)	76 ± 14	199 ± 51	88 ± 56	213 ± 42	91 ± 28	191 ± 62	74 ± 12	210 ± 60	72 ± 13	212 ± 81
minus value (after-before)	+123 ± 47^*∗*^		+125 ± 38^*∗*^		+100 ± 39^*∗*^		+139 ± 54^*∗*^		+139 ± 76^*∗*^	
Insulin (*µ*U/ml)	7.87 ± 3.13	1.49 ± 2.09	6.16 ± 2.30	1.35 ± 1.40	8.98 ± 5.71	3.86 ± 4.25	10.06 ± 2.89	1.00 ± 1.12	13.36 ± 9.52	1.74 ± 2.53
minus value (after-before)	−6.39 ± 2.61^*∗*^		−4.81 ± 2.32b^*∗*^		−5.11 ± 7.30 b		−9.06 ± 3.00^*∗*^		−11.63 ± 9.73a^*∗*^	
Glucose (mmol/l)	4.60 ± 0.37	4.59 ± 0.79	4.76 ± 0.35	4.59 ± 1.05	4.96 ± 0.33	4.59 ± 1.13	4.92 ± 0.44	4.50 ± 0.50	5.00 ± 0.41	4.58 ± 0.93
minus value (after-before)	−0.34 ± 0.69		-0.19 ± 0.72		−0.37 ± 0.83		−0.42 ± 0.46^*∗*^		−0.42 ± 0.85	
HOMA-IR	1.63 ± 0.25b	0.32 ± 0.17	1.32 ± 0.18b	0.31 ± 0.13	2.01 ± 0.50	0.91 ± 0.36 a	2.21 ± 0.24	0.21 ± 0.09 b	3.03 ± 0.85 a	0.41 ± 0.22
minus value (after-before)	−1.31 ± 0.21^*∗*^		−1.00 ± 0.18a^*∗*^		−1.10 ± 0.60a		−1.99 ± 0.25^*∗*^		−2.62 ± 0.87b^*∗*^	

All data are expressed as mean ± standard errors. ^*∗*^ indicates a significant difference (*P* < 0.05) after versus before each treatment considered independently (in bold). Two different letters (a, b) indicate a significant difference (*P* < 0.05) between treatments at a specific time (in bold and italic).

**Table 2:** Corrected small volume correction - (SVC-) based statistics on the brain blood-oxygen-level-dependent (BOLD) functional magnetic resonance imaging (fMRI) responses of minipigs to four types of electroacupuncture (EA) stimulation compared to control (no stimulation): sham (two bilateral sham points on the back and buttock), head-abdomen (cephalic Dafengmen #70 associated with abdominal three-locus Sanwan #35), back (bilateral Pishu #28 and Liumai #27), and leg (bilateral Housanli #63 and Hangou #79). A *p* value corrected with a Bonferroni correction at a threshold of 0.01 (peak level) was applied. The related uncorrected *p* value threshold after Bonferroni correction was consequently 0.00036 considering that 14 bilateral regions of interest (ROI) were selected *a priori*: nucleus accumbens (Acc), caudate nucleus (Cd), putamen (Pu), globus pallidus (GP), parahippocampal cortex (PHC), hippocampus (Hi), insular cortex (Ins), orbitofrontal cortex (OFC), anterior and dorsolateral PFC (aPFC and dlPFC), ventral and dorsal anterior cingulate cortex (vaCC and daCC), and ventral and dorsal posterior cingulate cortex (vpCC and dpCC). False-Wise Error (FWE) corrected *p* values are also indicated. The coordinates in the dorsal-ventral position related to the posterior commissure (in mm) are indicated as x, *y*, and z. The k-value represents the number of voxels per significant cluster. R: right; L: left.

		On—off					Off—on				
ROI	L/R	k	T	pFWE	p (uncor)	x, y, z	k	T	pFWE	p (uncor)	x, y, z
		Sham									
Pu	R						1	20.22	0.0692	0.0001	−9 5 16
	L										

Hi	R						21	32.92	0.0107	<0.0001	−11 10 −6
	L						2	20.53	0.0420	0.0001	5 9 0

aPFC	R						14	23.44	0.0479	<0.0001	−8 −2 34
	L						19	14.64	0.2499	0.0003	7 3 35

Ins	R										
	L						1	15.07	0.4857	0.0003	8 2 31

daCC	R						33	23.60	0.0366	<0.0001	0 4 35
	L						26	70.17	0.0014	<0.0001	1 3 34

dpCC	R						24	27.11	0.0390	<0.0001	−7 10 −10
	L										

PHC	R						35	53.38	0.0026	<0.0001	−12 0 −8
	L						10	16.41	0.0949	0.0002	6 10 −13

		Head-abdomen									
daCC	R										
	L	16	39.42	0.0084	<0.0001	2 9 26					
vaCC	R	2	15.75	0.0184	0.0003	0 13 9					
	L										

		Back									
daCC	R						31	37.74	0.0090	<0.0001	−4 11 24
	L										

dpCC	R						27	73.20	0.0019	<0.0001	−3 12 −11
	L										

		Leg									
dpCC	R										
	L	18	28.73	0.0327	<0.0001	1 20–5					
PHC	R										
	L	16	15.77	0.1069	0.0003	15 6–7					

**Table 3:** Corrected small volume correction- (SVC-) based statistics on the brain blood-oxygen-level-dependent (BOLD) functional magnetic resonance imaging (fMRI) responses of minipigs to three types of electroacupuncture (EA) stimulation compared to sham (false acupoints): sham (two bilateral sham points on the back and buttock), head-abdomen (cephalic Dafengmen #70 associated with abdominal three-locus Sanwan #35), back (bilateral Pishu #28 and Liumai #27), and leg (bilateral Housanli #63 and Hangou #79). A *p* value corrected with a Bonferroni correction at a threshold of 0.01 (peak level) was applied. The related uncorrected *p* value threshold after Bonferroni correction was consequently 0.00036 considering that 14 bilateral regions of interest (ROI) were selected *a priori*: nucleus accumbens (Acc), caudate nucleus (Cd), putamen (Pu), globus pallidus (GP), parahippocampal cortex (PHC), hippocampus (Hi), insular cortex (Ins), orbitofrontal cortex (OFC), anterior and dorsolateral PFC (aPFC and dlPFC), ventral and dorsal anterior cingulate cortex (vaCC and daCC), and ventral and dorsal posterior cingulate cortex (vpCC and dpCC). False-Wise Error (FWE) corrected *p* values are also indicated. The coordinates in the dorsal-ventral position related to the posterior commissure (in mm) are indicated as x, y, and z. The k-value represents the number of voxels per significant cluster. R: right; L: left.

		On—off					Off—on				
ROI	L/R	k	T	pFWE	p (uncor)	x, y, z	k	T	pFWE	p (uncor)	x, y, z
		Head-abdomen—sham									
Pu	R	12	21.51	0.0576	0.0001	−7 2 23					
	L	17	14.39	0.1944	0.0003	11 7 16					
Hi	R	16	16.53	0.0842	0.0002	−11 12 −5					
	L										
daCC	R	41	29.50	0.0188	<0.0001	−1 10 26					
	L										
PHC	R	4	42.02	0.0054	<0.0001	−1 10 26					
	L										

		Back—sham									
Cd	R										
	L	39	29.69	0.0256	<0.0001	6 8 15					
dlPFC	R						26	31.44	0.016613	<0.0001	−6 11 28
	L										
Ins	R	30	17.14	0.3164	0.0002	−13 10 19					
	L										
daCC	R	24	41.97	0.0065	<0.0001	0 1 36					
	L										
dpCC	R										
	L	16	24.53	0.0524	<0.0001	3 14 −12					
PHC	R	30	42.74	0.0052	<0.0001	−14 4 −10					
	L										

		Leg—sham									
Cd	R	43	52.95	0.0045	<0.0001	−7 11 10					
	L										
Pu	R										
	L	11	17.13	0.1159	0.0002	11 6 12					
Hi	R	28	21.32	0.0395	0.0001	−11 11 −4					
	L	59	32.54	0.0106	<0.0001	13 8 −6					
dpCC	R	79	17.14	0.1536	0.0002	−3 19 −7					
	L	31	21.37	0.0792	0.0001	3 17 12					

## Data Availability

The datasets for this study can be found in the INRAE open-access repository: https://doi.org/10.57745/HOMVKH.
